# Correspondence

**Published:** 1902-05-15

**Authors:** 


### CORRESPONDENCE.

*Editor Dental Register:*—In the March number of the REGISTER appears a reference to pink teeth. This condition is not very common but I have seen at least ten or twelve cases. In one family the history of pink or rather almost red incisors, ran through many generations. There seemed to be no other peculiarity of development, all the family had dark hair, brown eyes, dark skin, and rather highly colored cheeks. In two boys of another family only the upper centrals were pink but not as bright a pink as in the other family mentioned. It is undoubtedly a case of peculiarity of pigmentation of the enamel as appeared from a rather careless examination of some of the teeth after they were cut off in preparation for crowns that were made to harmonize with the other teeth.

Toronto, Ontario. *A. E. Webster.*

Dr. Blackmarr, of Jackson, Michigan, thinks this phenomenon is due to an unusually large pulp and thin enamel and dentin coverings.—Ed.

Since commenting on this case we have had referred to us a young lady whose teeth have a decidedly dark-brown pigment, and they have always been so. A similar pigmentation also appears in the mouth of an aunt. The pulps in the teeth are in a normal condition, but the color of the teeth is such as to greatly disfigure a very pretty face. This discoloration is so distasteful to the young lady that she has consulted several dentists in the hope that some one would be able to either bleach the teeth or would be willing to cut off the crowns and replace them with more harmonious artificial ones. This discoloration is unusual and can not be accounted for on the theory advocated by Dr. Blackmarr.

---

*Editor Dental Register:*—Can the pulp be removed from the first deciduous molar of a child of four years without detriment to the permanent tooth? Also is cocain injection of one and one half percent solution, contraindicated for a patient who has been drinking? Does an abscessed condition preclude the use of cocain in extracting? *J. A. Heidbrink.*

A suppurating pulp in a deciduous tooth even at the age of four years had probably better be removed. It should be done however surgically under anesthesia by cocain injection or pressure, and the canal sterilized and filled antiseptically. An arsenical application might by apical infiltration involve the peridental tissues to the injury of the permanent tooth.

Cocain should not be administered to a patient who is sufficiently under the influence of alcoholics to be nervously excited, neither should it be given to one who is depressed by an over dose of it. There is no reason, however, why it may not be used in connection with a tonic or stimulating dose of whisky, for instance. As alcoholics affect people very differently it will be wise to carefully note the condition of the patient as to mental and reflex excitability, as well as to the affect produced by the alcohol on the respiration and circulation. *Never* use over a *one-percent* solution of cocain for any hypodermic injection about the teeth. Never inject cocain or anything else into abscessed tissues for fear of forcing the absorption of the pus by the blood vessels and the production of systemic pyemia. In such cases always inject the peridental membrane and not the gum tissues. Use a small needle and pass it into the membrane close to the root of the tooth. This may sometimes be facilitated by making a seat for the syringe point by drilling a very small hole along the side of the root with a small Gates-Glydden drill rapidly revolved by the dental engine.

